# Training and Maintaining System-Wide Reliability in Outcome Management

**DOI:** 10.1007/s10826-012-9694-x

**Published:** 2012-12-01

**Authors:** Melanie A. Barwick, Diana J. Urajnik, Julia E. Moore

**Affiliations:** 1 Child Health Evaluative Sciences, Research Institute, The Hospital for Sick Children, 555 University Avenue, Toronto, ON M5G 1X8 Canada; 2 School of Rural and Northern Health, Laurentian University, Sudbury, ON Canada; 3 Li Ka Shing Knowledge Institute, St. Michaels Hospital, Toronto, ON Canada

**Keywords:** Outcome management, Rater reliability, Train-the-trainer, CAFAS, Rater drift

## Abstract

The Child and Adolescent Functional Assessment Scale (CAFAS) is widely used for outcome management, for providing real time client and program level data, and the monitoring of evidence-based practices. Methods of reliability training and the assessment of rater drift are critical for service decision-making within organizations and systems of care. We assessed two approaches for CAFAS training: external technical assistance and internal technical assistance. To this end, we sampled 315 practitioners trained by external technical assistance approach from 2,344 Ontario practitioners who had achieved reliability on the CAFAS. To assess the internal technical assistance approach as a reliable alternative training method, 140 practitioners trained internally were selected from the same pool of certified raters. Reliabilities were high for both practitioners trained by external technical assistance and internal technical assistance approaches (.909–.995, .915–.997, respectively). 1 and 3-year estimates showed some drift on several scales. High and consistent reliabilities over time and training method has implications for CAFAS training of behavioral health care practitioners, and the maintenance of CAFAS as a global outcome management tool in systems of care.

## Introduction

Outcome management systems are rapidly becoming a core component of healthcare services (e.g. Knaup et al. 2009). As policy directives drive increased accountability for effective and efficient services, providers are expanding their efforts to implement evidence-based practices, and the outcome management systems that necessarily support them. In the absence of systematic outcome management, providers cannot determine either the quality of their implementation efforts, or related client outcomes (Durlak and DuPre 2008).

Outcome management receives support from earlier research showing that the general trajectory of change in successful therapy is predictable (e.g. Howard et al. 1996). Outcome measures can be used to determine the appropriateness of a treatment plan, the need for further treatment, and can serve as a key indicator of treatment progression or lack thereof (Howard et al. 1996). This latter point is further strengthened by Whipple et al. (2003), who found that clients at risk for a negative outcome were less likely to deteriorate, more likely to stay in treatment longer, and twice as likely to achieve a clinically significant change when their therapists had access to outcome and alliance information.

Quality improvement efforts in children’s mental health emphasize the inclusion of outcome measurement as a matter of routine care (e.g. Garland et al. 2010). For example, Bickman (2008) describes how the use of measurement feedback systems (MFS) may enhance clinical practice and the quality of service delivery. Measures of clinical status (e.g. symptoms, functioning) and process (e.g. practice elements, therapeutic alliance), ongoing monitoring concurrent with treatment, and feedback to practitioners are key features of these systems. Continual feedback is critical, as it may optimize intervention effectiveness. In this manner, the tracking of individual progress, fidelity to protocol, and organizational program results, can be used to inform and maximize the benefits of treatment (Schmidt 2012; Chorpita et al. 2008). Importantly, research suggests that objective feedback may improve child outcomes (e.g. Warren et al. 2009).

In Ontario, Canada, the provincial government mandated the assessment of functional outcomes in 2000 for children ages 6-to-17 years of age who receive mental health services in one of 120 organizations situated throughout the province. The tool selected for outcome measurement was the Child and Adolescent Functional Assessment Scale (CAFAS) (Hodges 2003; Raphael et al. 1999). Technical assistance (e.g. training) for the CAFAS is the responsibility of a team of health services researchers, educators, and data analysts (*CAFAS*-*In*-*Ontario*) at The Hospital for Sick Children in Toronto. Over the last decade, the team has developed strategies to support CAFAS implementation (Barwick et al. 2002), the use of the tool with Aboriginal children (Barwick et al. 2004), and for utilizing outcome data to meet standards of accreditation for service organizations (Accreditation Working Group, Children’s Mental Health Ontario 2004). Important considerations arising from implementation concern the efficiency of practitioner training and re-certification, and the utility of a train-the-trainer approach to establish internal technical assistance for the system.

### Child and Adolescent Functional Assessment Scale (CAFAS)

The CAFAS is a practitioner-rated measure of functional impairment in children (6–17 years of age) who have, or may be at risk for emotional, behavioural, substance use, or psychiatric problems (Hodges 2003). It contains a “menu” of behavioral descriptors or items divided into eight subscales: School/Work, Home, Community, Behavior Towards Others, Moods/Emotions, Self-Harmful Behavior, Substance Use, and Thinking Problems. Ratings are also generated for the child’s caregiver on two additional scales, Material Needs and Family/Social Support, to assess the caregivers’ ability to provide for the child within these domains. For each scale, the practitioner selects the item that best describes the most severe level of dysfunction for the time period specified (e.g. the last month). Impairment levels are assigned quantitative values for generating continuous scores: Minimal or no impairment (0), Mild impairment (10), Moderate impairment (20), and Severe impairment (30). There are no cut-off scores, but rather, a general framework derived from research with the CAFAS (Hodges and Wong 1996; Hodges et al. 1997). The scale scores are combined to form a total score (0–240) that reflects overall functional impairment.

The CAFAS is a well-established measure used for system-wide outcome monitoring (e.g. Hodges and Wotring 2004); and by child serving agencies in mental health, juvenile justice, child welfare, and education (e.g. Friesen et al. 2003; Lyons et al. 2003; Vernberg et al. 2008). However, use of the tool requires certification using the self-training manual developed by the test author (Hodges 2006). The manual contains detailed scoring information, demonstration, and testing vignettes. The vignettes comprise disguised, actual clinical cases, and provide the basis for certification ratings. Procedures are standardized to ensure that all practitioners use the same rules and definitions of terms. To become a reliable rater—or certification—involves training to criteria. In other words, the achievement of “reliability” entails concordance with a criterion score, or gold standard rating for each subscale. Each criterion was derived through consensus scoring, undertaken by the CAFAS author and a board certified child psychiatrist (Hodges and Wong 1996).

Pearson correlations of ≥.70 (Mood, Self-Harm scales), ≥.85 (School, Home, Community, Behaviour), and ≥.90 (Total score) with the standard for each scale are the criteria for CAFAS reliability (Hodges 2005). The calculated reliabilities represent, in effect, measures of consistency (e.g. with criterion scores). Concordance data, however, is distinguished from that of inter-rater reliability. Inter-rater reliability is a measure of consistency or agreement between different raters, and is typically estimated by the intraclass correlation coefficient (ICC) (Kaplan and Saccuzzo 1997). Research with the CAFAS supports both the criterion-related validity (e.g. Fallon et al. 2006; Hodges et al. 1999; Manteuffel et al. 2002) and inter-rater reliability (e.g. Hodges and Wong 1996; Ogles et al. 1999) of the measure.

The maintenance of reliability or accuracy is critical, perhaps particularly so for outcome measures based on cognitive or performance ratings. Inconsistencies interfere with data quality by introducing additional variance; a common source of inconsistency is rater drift (Wilson and Case 2000). Rater drift occurs when raters exhibit different effects over time, or drift from standard levels by unintentionally redefining criteria (e.g. scoring rules) (Kaplan and Saccuzzo 1997). For the CAFAS, it is recommended that certified raters re-establish reliability annually, or every 2 years to control for such inconsistency. Training is similar to that for initial certification, and requires the completion of booster vignettes in order to re-certify. However, little is known about how fidelity to the scoring standards may vary with time. Few studies have quantified rater drift using the CAFAS (e.g. Franco et al. 2002), despite the necessity of addressing drift through adequate training methods.

### Outcome Management in Ontario

The CAFAS has been used for outcome management in approximately 120 children’s mental health organizations in Ontario since 2000; the number of organizations has fluctuated slightly from year to year due to amalgamations. Provincially, the CAFAS has supported the assessment of treatment effectiveness, helped to standardize the measurement of quality within and between organizations, and has contributed to the development of an infrastructure to support and improve service delivery across the system. Implementation requires use of the electronic version of the tool (Version 5.4), and reliability certification. The initial phase of reliability training took place between 2000 and 2003. In 2004, training on the electronic CAFAS began, and to date, 6,742 practitioners have been trained.

The *CAFAS*-*In*-*Ontario* technical assistance (implementation) team provides training to practitioners (e.g. child and youth workers, social workers, psychologists, psychiatrists) for provincially-funded organizations. The external technical assistance training is standardized using the CAFAS self-training manual (Hodges 2006), and 2-day, face-to-face group workshops. Supplemental assistance and support are provided where necessary until practitioners become reliable in the use of the measure. However, user organizations may train their own personnel via a designated trainer. In this manner, the implementation team also provides train-the-trainer workshops to certify practitioners as internal trainers, who then take on CAFAS reliability training of others within their organization (i.e., internal technical assistance). Train-the-trainer certification is based on: (1) the manual for training coordinators, clinical administrators, and data managers (Hodges 2005); (2) train-the-trainer workshop attendance; and (3) reliability certification.

Best practice in Ontario involves using the CAFAS to assess progress, to assist with assessment, formulation and planning, and to measure overall outcomes. Organizations are expected, at a minimum, to complete ratings as close to treatment entry and discharge as possible. Practitioners are also encouraged to use the tool periodically (e.g. at 3 month intervals, scheduled review times) while the client is receiving service, in order to gauge treatment response. Aggregate provincial data has been used to inform organization- and system-level decision-making. However, the success of the initiative depends, in part, on the reliability and validity of the measurement effort, which can be assessed via rater consistency with the gold standard ratings for the tool. Currently, in Ontario, reliable raters are required to complete annual booster exercises to re-certify. Re-establishing reliabilities can be done electronically, but requires administrative support to score vignettes and provide remedial support. Hence, to do so every 2 or 3 years would be more efficient. Thus, our first objective was to evaluate CAFAS scores against standard ratings, and changes at 1- and 3-years post initial training. We were interested in potential rater drift. Results would inform the timing and necessity of follow-up reliability certification.

With CAFAS use spanning the province’s system of care, reliability training also needs to be feasible and cost-effective. One way to achieve this is to explore alternative training methods. We developed and implemented a CAFAS train-the-trainer workshop (i.e., internal technical assistance) to bolster system-wide sustainability of the tool. Financial expenditures (e.g. travel, trainer accommodation) for the initial provincial training period were 15 times greater for external technical assistance (external TA) versus internal technical assistance (internal TA). As such, our second objective was to empirically establish the utility of the more economical internal TA approach.

This study sought to (1) examine initial CAFAS reliabilities for each training method (external TA vs. internal TA); (2) rater drift over a 1- and 3-year period following achievement of initial reliability; and (3) to compare reliability and rater drift for the two training methods. We hypothesized that drift would be evident in both trainee groups because it is a common finding for psychological tools used in clinical practice (Maruish 2004). The findings of Franco et al. (2002) suggest that reliabilities would be slightly higher for practitioners trained by an external TA approach as compared to those trained by an internal TA approach. Our intention was to establish an acceptable time interval for re-certification training, and to determine the comparability of training methods.

## Method

### Sample

Practitioners were selected from an overall pool of 2,344 individuals (71 children’s mental health organizations) who had achieved reliability on the CAFAS in 2000–2003. Of the 2,344 practitioners, 2,204 (94 %) had been trained by *CAFAS*-*in*-*Ontario* technical assistance trainers. A sample of 315 trainees (“external TA” group) was selected according to the nine provincial regions (n = 35 from each of Northern, North East, Central East, Eastern, South East, Toronto, Central West, South West, and Hamilton/Niagara areas). Also of the 2,344 practitioners, 140 (6 %) were trained by an internal trainer (“internal TA” group). These trainees comprised all individuals who had been trained internally during the specified time period. Study procedures were approved by the Research Ethics Board (REB) of The Hospital for Sick Children.

### Procedure

#### Reliability Training

##### External Technical Assistance

CAFAS rater reliability training for Ontario was developed using the measure’s self-training manual (Hodges 2006) and experience gained in an implementation feasibility study (Boydell et al. 2004). A 2-day workshop was developed based on practitioner feedback from the study. Three external TA trainers (all master’s level clinicians with 5–20 years experience in child and youth mental health) travelled across the province conducting training workshops. The workshops included information about Ontario’s outcome measurement initiative, and an in-depth review of the CAFAS measure and scoring rules. Six demonstration vignettes were completed, with discussion of the scoring rules. On the second day of training, practitioners individually scored ten reliability vignettes, working at their own pace. Trainees were permitted to discuss scoring issues and difficulties with the trainer on an individual basis.

##### Internal Technical Assistance

Reliability training for practitioners trained by internal trainers followed the same procedure as that for practitioners trained by the external technical assistance trainers. In this manner, training also entailed participation in a 2-day workshop that began with an overview of the measurement initiative, instruction on the CAFAS scoring rules, and completion of six demonstration vignettes, followed by discussion of the rules. Practitioners also completed ten case vignettes on the second day of training.

All internal TA trainers had received train-the-trainer certification by the external technical assistance team. This certification required participation in a separate, 2-day training workshop uniquely for those wishing to train CAFAS reliability for practitioners within their organization. The manual for training coordinators, clinical administrators, and data managers (Hodges 2005) provided the basis for certification; internal TA trainers were reliable raters themselves on the CAFAS. Train-the-trainer sessions involved a review of the CAFAS reliability requirements, scoring, and case vignettes. Additional instruction focused on the procedures (e.g. reliability workshop) and technical aspects (e.g. required materials) of training practitioners on-site.

#### Establishing Reliability

Initial reliability training involved attendance at the 2-day workshop sessions, for both internal and external TA practitioners. Session size varied in terms of ratio of trainer to trainees. Sessions for the external technical assistance sample included 7–29 individuals, with an average session size of 18. However, the mean group size was 2 (range = 1–8) for individuals trained with the internal TA approach. Group size depended on the unique needs of organizations, and was affected by such issues as staff turnover, and the number of new practitioners that required training; thus the smaller sessions.

Reliability was established by rating the 10 case vignettes provided in the self-training manual (Hodges 2006). In Ontario, reliability is defined as demonstrating 80 percent agreement with the gold standard criterion on each of the 10 CAFAS subscales for 10 case vignettes. Each vignette detailed an individual child’s case history (e.g. symptoms, behaviour, family dynamics), and included clinical data, such as that from structured diagnostic interviews, and information from multiple sources, such as from parents and teachers.

Ratings were evaluated by session trainers; reliability was indicated by two or fewer errors on each subscale for each vignette (Hodges 2005). Practitioners not achieving reliability were given the opportunity to complete supplemental vignettes. All trainees were then contacted one and 3 years after attainment of initial reliability via electronic mail, and asked to complete 10 booster case vignettes at both time points. Data were thus collected in a prospective manner; trainees were identified at initial certification, with follow-up in order to implement the 1- and 2-year periods between re-certification.

### Data Analysis

Reliability ratings yielded subscale scores (0, 10, 20, 30) for the 10 case vignettes. The score for each scale was compared to its’ corresponding criterion score (the “gold standard” rating of the CAFAS developer; Hodges 2005). Pearson product-moment correlations were calculated and averaged across raters in each trainee sample. Analyses were conducted for the eight child subscales, and the Total score for the initial, 1-, and 3-year reliability exercises. Data were not analyzed for the two caregiver domains (Material Needs and Family/Social Support), as these scales are not included in the Total score.

Chi square and analyses of variance (ANOVAs) were used to compare the demographic characteristics of practitioners trained externally and internally. To assess changes in scale reliabilities (correlations with the criterion) over time, repeated-measures ANOVAs were computed separately for each trainee group. Time (initial, 1-, 3-year) was specified as a within-subject factor. Finally, training method (internal vs. external TA) was included as a between subject factor in a final repeated measures model. Of interest, was whether reliabilities were dependent upon method of instruction; a time by group interaction term was also entered into the analysis.

## Results

### Descriptive Statistics

Demographic characteristics for the two trainee samples are presented in Table 1. Information for practitioners not included in the study (n = 1,889) is also shown. External TA trainees had more years experience in the field, on average, than those in the population and internal TA samples (F_2,2025_ = 15.9, *p* < 0.001). In addition, a larger percentage of these practitioners (41 %) held graduate or professional degrees (e.g. M.S.W., Ph.D., M.D.) (χ^2^ = 11.2, *p* < 0.05) and senior/supervisory positions (e.g. managers) (χ^2^ = 10.8, *p* < 0.01) than other degree/job types as compared to the population and internal TA practitioners. The internal TA sample included significantly more females (87.2 %) (χ^2^ = 8.6, *p* < 0.05).

**Table 1:** Demographic characteristics of raters by sample

Characteristic	CAFAS practitioner population^1^ (n = 1,889)	External TA (n = 315)	Internal TA (n = 140)
Mean years experience (SD)***	12.4 (8.2)	14.3 (8.0)	9.2 (7.1)
Education level (%)*			
College (e.g. SSW, CYW)	34.5	36.3	34.3
Undergraduate (e.g. BSW, BSc)	31.3	22.7	35.3
Graduate/professional (e.g. PhD)	34.2	41.0	30.4
Job description (%)**			
Clinician	77.3	72.0	87.8
Senior/supervisory	22.7	28.0	12.2
Gender (% female)*	76.1	76.8	87.2

^1^Practitioners not included in the study; these individuals had achieved initial reliability and were from the same 71 organizations as external and internal TA trainees

* *p* < .05, ** *p* < .01, *** *p* < .001

Sample sizes decreased over time. Of the 1,889 CAFAS certified practitioners, 1,158 (61.3 %) had completed the 1-year booster exercise, and 695 (36.8 %), the 3-year booster. Of the 315 external TA trainees with initial certification, all were retained at 1-year. However, 122 were lost to follow-up at 3 years (final n = 193, or 61 %). Seventy-one (51 %) internal TA practitioners had a 1-year re-certification; 32 (23 %) were retained at 3-years.

Analyses comparing retained versus non-retained trainees, showed that external TA practitioners lost to follow-up had a higher level of education than those with a 3-year re-certification (*p* = .03). In contrast, internal TA practitioners retained at the 1-year point had more education than those who had dropped out (*p* = .04). Due to different rates of retention for training method and demographic variations by sample, further analyses were adjusted for years of experience, gender, education, job and region.

### Reliability and Rater Drift

#### External Technical Assistance

High mean correlations with the criterion were found for initial, 1- and 3-year reliabilities on all CAFAS subscales for practitioners trained by the external technical assistance team (Table 2). Average correlations for initial reliabilities ranged from .911 (Mood) to .994 (Self-Harm). 1-year reliabilities ranged from .885 (Behaviour) to .995 (Substance Use). Reliabilities achieved 3 years after initial certification, e.g. second follow-up exercise, were also high (.916 for Mood to .992 for Substance Use).

**Table 2:** Pearson correlations between external TA trainee (n = 315) scale scores and criterion for initial reliability, 1- and 3-year re-certification

Scale	Initial (SD)	1-year (SD)	3-year (SD)	*F*	LSD
School	.956 (.07)	.971 (.07)	.982 (.04)	8.84***	1 year, 3 year > I
Home	.980 (.03)	.972 (.04)	.973 (.05)	2.10	
Community	.973 (.06)	.958 (.06)	.986 (.06)	10.28***	3 year > I, 1 year; 1 year < I
Behaviour	.953 (.06)	.885 (.17)	.945 (.06)	22.21***	I, 3 year > 1 year
Mood/emotions	.911 (.08)	.953 (.05)	.916 (.15)	10.0***	1 year > I, 3 year
Self-harm	.994 (.03)	.970 (.06)	.977 (.06)	10.77***	I, 3 year > 1 year
Substance use	.987 (.04)	.995 (.03)	.992 (.03)	3.31*	1 year > I
Thinking	.983 (.05)	.963 (.06)	.962 (.05)	10.63***	1 year, 3 year < I
Total	.990 (.01)	.989 (.01)	.986 (.02)	5.07**	3 year < I

Based on valid cases for analyses; initial = 315; year 1 = 315; year 3 = 193

* *p* < .05, ** *p* < .01, *** *p* < .001

*LSD* Fisher’s least significant difference

Reliability estimates changed over time (see Table 2). Correlations with the criterion were higher at one (*p* = .033) and 3 years (*p* < .001) than at initial reliability for the School subscale. Reliability was also higher at 3 years for the Community scale compared with initial (*p* = .042) and 1-year (*p* < .001) coefficients. Correlations were highest at 1-year re-certification for the Mood (*p* with initial <.001; *p* with 3-year = .001) and Substance Use (*p* with initial = .011) scales.

Reliabilities drifted from initial certification to one (*p* < .001) and three (*p* < .001) years for the Thinking scale. Reliability was lowest at 1-year for both the Behaviour (*p* with initial <.001; *p* with 3-year <.001) and Self Harm (*p* with initial < .001; initial and 3-year *p* = .001) scales. Consistency with the criterion decreased from initial- to 3-year re-certification for the CAFAS Total score (*p* = .002). No statistically significant changes were observed for the Home scale coefficients.

#### Internal Technical Assistance

Average correlations with the criterion were also high for practitioners trained by the internal TA approach. Coefficients ranged from .933 (Mood scale) to .998 (Self-Harm) at initial certification, and .919 (Behaviour) to .998 (Substance Use) at the time of 1-year re-certification (Table 3). Reliabilities obtained upon completion of the second follow-up (at 3-years) were consistently high, ranging from .890 (Mood) to .997 (Community).

**Table 3:** Pearson correlations between internal TA trainee (n = 140) scale scores and criterion for initial reliability, 1- and 3-year re-certification

Scale	Initial (SD)	1-year (SD)	3-year (SD)	*F*	LSD
School	.937 (.11)	.989 (.02)	.960 (.06)	3.46*	1 year > I, 3 year
Home	.966 (.04)	.965 (.05)	.959 (.04)	0.23	
Community	.968 (.06)	.981 (.04)	.997 (.01)	4.35*	3 year > I, 1 year
Behaviour	.943 (.09)	.919 (.08)	.947 (.06)	1.10	
Mood/emotions	.933 (.05)	.939 (.05)	.890 (.16)	2.11	
Self-harm	.998 (.01)	.976 (.04)	.953 (.09)	4.78*	1 year, 3 year < I
Substance use	.984 (.06)	.998 (.01)	.996 (.01)	1.68	
Thinking	.987 (.03)	.964 (.06)	.980 (.04)	3.15	
Total	.989 (.01)	.992 (.01)	.984 (.01)	3.43*	3 year < 1 year

Based on valid cases for analyses; initial = 140; year 1 = 71; year 3 = 32

* *p* < .05, ** *p* < .01, *** *p* < .001

*LSD* Fisher’s least significant difference

Reliabilities improved with time for the Community subscale. The correlation at 3-years was higher than those achieved at both initial (*p* = .025) and 1-year (*p* = .043) certification points. Coefficients also increased for the School scale, with the highest observed at 1-year (vs. initial, *p* = .024; vs. 3-year *p* = .002).

Reliability decreased from .998 (initial) to .976 at 1-year (*p* = .002) for Self-Harm; the 3-year estimate showed the greatest change from initial certification (*p* = .011). The lowest coefficient for the Total CAFAS score was at 3 years (*p* = .021 between 1- and 3-years). There were no significant changes in correlations over time for the Home, Behaviour, Mood, Substance Use, or Thinking scales.

### Training Method

Repeated measures analysis with group as a between subject factor was used to examine whether changes in reliabilities depended on training method. Results indicated no main effects for method on seven of the eight subscales, or for the CAFAS Total score (all F > .05) (Table 4). However, group differences on the Home scale approached significance (F_1,188_ = 3.54, *p* = .06, partial eta^2^ = .02). Reliability was higher for clinicians trained by an external TA trainer (M = .975) as compared to those trained by an internal TA trainer (M = .964). A method by time interaction was found for the Self Harm scale (F_2,386_ = 3.21, *p* < .05, partial eta^2^ = .02). Polynomial contrasts indicated an interaction for the linear component of time (F_1,188_ = 6.56, *p* < .05). There was more drift from initial- to 3-year reliabilities for internal TA, versus external TA trainees. There were no significant interactions between training method and time on the School, Home, Community, Behaviour, Mood, Substance Use, or Thinking scales; nor for the CAFAS Total score (all F > .05).

**Table 4:** Initial, 1- and 3-year re-certification reliabilities: main effects of time and training method, and interactions between time and training method

Scale	External TA			Internal TA			Time	Training	Time by training
	Initial (SD)	1-year (SD)	3-year (SD)	Initial (SD)	1-year (SD)	3-year (SD)	*F*	*F*	*F*
School	.958 (.07)	.972 (.07)	.981 (.04)	.943 (.09)	.989 (.02)	.965 (.06)	4.70*	0.92	1.63
Home	.980 (.03)	.972 (.04)	.973 (.05)	.975 (.03)	.963 (.05)	.958 (.04)	1.71	3.54^a^	0.29
Community	.973 (.06)	.960 (.06)	.987 (.06)	.973 (.07)	.985 (.04)	.998 (.01)	2.70	1.64	0.76
Behaviour	.952 (.06)	.898 (.14)	.947 (.05)	.958 (.07)	.922 (.08)	.940 (.07)	4.41*	0.34	0.50
Mood/emotions	.908 (.08)	.952 (.05)	.918 (.15)	.932 (.05)	.939 (.04)	.913 (.13)	1.92	0.01	0.71
Self-harm	.994 (.03)	.974 (.05)	.979 (.06)	.999 (.01)	.979 (.03)	.947 (.10)	8.15***	1.17	3.31*
Substance use	.986 (.04)	.995 (.03)	.993 (.03)	.980 (.07)	.999 (.01)	.995 (.01)	2.86	0.25	0.41
Thinking	.983 (.05)	.964 (.06)	.965 (.05)	.989 (.02)	.969 (.04)	.978 (.04)	3.11*	0.18	0.14
Total	.990 (.01)	.990 (.01)	.987 (.02)	.991 (.01)	.992 (.01)	.985 (.01)	3.97*	0.01	0.53

Adjusted for clinician years of experience in the field, gender, level of education and job, region

* *p* < .05, ** *p* < .01, *** *p* < .001

^a^Marginally significant at *p* = .05

Main effects were found for time on the School (F_2,386_ = 4.70, *p* < .05, partial eta^2^ = .02), Behaviour (F_2,386_ = 4.41, *p* < .05, partial eta^2^ = .02), Self Harm (F_2,386_ = 8.15, *p* < .001, partial eta^2^ = .04), and Thinking (F_2,386_ = 3.11, *p* < .05, partial eta^2^ = .02) scales, and for the CAFAS Total score (F_2,386_ = 3.97, *p* < .05, partial eta^2^ = .02). In other words, coefficients varied as a function of time regardless of training method on these scales.

For the school scale, correlations with the criterion were higher at one (M = .980) and 3 years (M = .972) than at initial reliability (M = .947) (initial vs. 1-year, *p* = .006; initial vs. 3-year, *p* = .014). Reliabilities decreased for the Self Harm scale (initial M = .997, 1-year M = .977, 3-year M = .961); reliabilities at each re-certification were lower than that initially achieved (initial vs. 1-year, *p* = .003; initial vs. 3-year, *p* < .001). Likewise for the CAFAS Total score (initial M = .991, 1-year M = .990, 3-year M = .986) (*p* = .025 and *p* = .028 for initial vs. 3-year, and 1- vs. 3-year, respectively).

Reliabilities for Behaviour (initial M = .954, 1-year M = .910, 3-year M = .945) and Thinking (initial M = .984, 1-year M = .964, 3-year M = .971) also tended to drop over time. Correlations were lowest at 1-year for both scales (Behaviour initial vs. 1-year, *p* = .015; 1-year vs. 3-year, *p* = .05; Thinking initial vs. 1-year, *p* = .010). There were no effects for time on the Home, Mood, or Substance Use scales (all *p* > .05).

## Discussion

Functional impairment measures have been shown to have utility in both treatment planning and monitoring client progress (Maruish 2004). CAFAS is widely used in the United States, in 48 states and over 2,500 state and county level institutions, including the Departments of Social Services, Centers of Community Health, Juvenile facilities, hospitals, alternative schools, and child welfare centres (Multi-Health Systems Inc., personal communication, May 22, 2012). In Canada, CAFAS is used in 65 provincial, municipal, and private institutions in 5 provinces (AB, BC, MB, ON, SK). Considering the widespread use of the CAFAS across Canada and the United States (e.g. Barwick et al. 2004; Boydell et al. 2004; Fallon et al. 2006; Hodges and Wotring 2000; Hodges et al. 2004; Manteuffel et al. 2002; Roy et al. 2008), and the cost of maintaining a system of care, practitioner training needs to feasible and economical. For this reason, we examined our reliability certification procedures and methods of training. We sought to determine if reliability, as assessed via concordance agreement, would drift over time. To our knowledge, this is one of only two studies (Franco et al. 2002) to examine this issue with the CAFAS, and the first to compare reliabilities for different training methods.

Results showed consistently high correlations with the standard ratings. Only one subscale coefficient was below .90, and of the 48 reliabilities that were calculated, 79.2 % were above .95. All coefficients for the Total score were above .98. Hodges and Wong (1996) have reported comparable Total estimates (.92–.96) using vignettes and Pearson correlations for agency staff. Coefficients for individual scales ranged from .90 (School/Work) to .98 (Substance Use). However, the Hodges study only reports reliabilities for ratings completed at one point in time. Nevertheless, our analyses showed no effects for time, or statistically significant changes in reliabilities, for four of the eight subscales (Home, Community, Mood, or Substance Use). Of the remaining four, coefficients for the School/Work scale improved; correlations with the criterion were higher at the time of both follow-up exercises than at initial certification. This is contrast to the Self Harm, Behaviour, and Thinking scales, for which reliabilities tended to decrease.

There may be several possible explanations for the observed drift. First, drift may have occurred on scales for which practitioners had little experience, if they encountered few clients with the associated condition. This might explain the results for the Self-Harm and Thinking scales (e.g. lower frequency or organicity for these conditions). Second, and alternatively, more experience with functional impairment on these scales (e.g. with severely depressed youth), may lead to under-estimation, for children with less severe impairments. Last, other rater characteristics, such as education, or gender, may have influenced the results.

Of interest in the current study, was the comparability of our training methods. We hypothesized that external TA trainees would evidence less drift than those trained with the internal TA approach, based on a study conducted by Franco et al. (2002). For example, these authors demonstrated that raters with the least drift were more likely to be trained by the national evaluation of The Comprehensive Community Mental Health Services for Children and Their Families Program, as opposed to self-training. Yet our results showed few differences between trainee groups in final models. Only two statistically significant effects were found. Reliability was marginally higher for the external TA group on the Home scale. There was also a group by time interaction for Self-Harm; internal TA practitioners drifted more on this scale than did external TA trainees. In general, coefficients varied as a function of time, regardless of training method (main effect for four subscales, and Total).

That few effects were found for training method, may suggest that there are genuinely no differences in the fidelity of ratings for externally trained versus internally trained practitioners. Both groups of trainees had comparably high reliabilities, for initial, 1- and 3-year certification ratings. The results of separate analyses for each group showed some drift on similar subscales, e.g. Self-Harm, Total score. However, differential attrition, and the relatively small amount of follow-up data for the internally trained practitioners may have influenced our findings, and thus the interpretability of method differences.

### Limitations

Several aspects of this study warrant further consideration. First, sampling issues may have affected our results. Practitioners were from an overall pool of individuals who had achieved reliability on the CAFAS during initial implementation. Externally trained practitioners were selected from each of the nine provincial regions; we sought to obtain a system-wide sample that was trained by the implementation team. On the other hand, the internal TA group comprised all individuals available during the same period. However, there is some question as to whether trainees were representative of all practitioners who had achieved reliability. Descriptive analyses showed demographic variations by sample. Externally trained practitioners had more experience in the field, and higher levels of education than both the population and internal TA groups. More internally trained practitioners were female. Furthermore, as mentioned, there was differential attrition. The attrition rate was larger for internally trained practitioners, which likely impacted both the equivalency of groups over time, and the power to detect effects for training method. Although analyses were adjusted for demographic factors, further work that examines the potential moderating effects of practitioner characteristics (e.g. education, gender) is required.

A second issue, concerns the specificity of our results. We relied on written vignettes versus the use of actual clinical cases. In this regard, the vignettes were standardized cases used for training purposes. Here, achievement of reliability was the goal of sessions, which entailed 80 % agreement with the standard criterion ratings. This may explain the consistently high reliabilities across scales and training method, and the small changes (albeit with some statistical significance) with time. For example, training-to-criteria would likely produce higher estimates, than those obtained using archival cases. Ogles et al. (1999) found that reliabilities were significantly higher for vignettes (0.90) than for case data (0.66). Moreover, prior experience with the CAFAS, the number of assessments completed, or the additional, re-certification training, may have resulted in higher quality ratings (Schorre and Vandvik 2004).

Despite the possibility of overestimation, and little variability in reliabilities, our results are meaningful from a training perspective. The use of vignettes provided a standardized approach to our training protocol and study procedures. The findings provide some support for an internal TA model, and 3-year instead of annual certification. Nevertheless, we concede that results may not generalize to actual ratings in clinical practice. Furthermore, given the relatively large number of statistical tests conducted, sample sizes, and unclear patterns of change, effects for time may be spurious. Overall, reliabilities were stable from the time of initial reliability through to the follow-up assessments. The replication of our study with new samples of practitioners is required.

Last, this study does not account for training session factors that could, conceivably, have influenced our results. Session size varied between external and internal TA trainees. The external TA team conducted workshops for upwards of 29 practitioners, whereas sessions for internal TA instruction were comparatively smaller, with an average of two individuals per session. In the latter case, group size depended on the unique needs of organizations, e.g. number of new practitioners that required training. Whether session size influenced the quality or intensity of instruction, and subsequently, practitioner ratings, is not known. Likewise, trainer adherence to protocol was not assessed; yet could have varied by trainer, and organization. Future studies would benefit from the inclusion of process measures (e.g. in-session adherence), that assess fidelity to training protocol (Proctor et al. 2011).

### Implications

This study has implications for CAFAS use in outcome management, and the training of practitioners to this end. Examined, were reliability certification and two methods of instruction. Results suggest the: (1) adoption of an alternate time interval for re-certification; and (2) continued use of an internal TA approach. Practitioners were able to maintain a high degree of consistency with the criterion ratings over time. There were also few differences between our external TA and internal TA trainees.

We concur with Franco et al. (2002) that effective training materials and workshops are key ingredients to maintaining consistent reliability, and suggest that the rigor of our internal TA model led to the largely equivalent reliabilities between training methods. However, as there was drift on some scales (e.g. Self-Harm, Thinking), future research should assess how additional material, or alternate strategies (e.g. communities of practice, web-based learning forums) could help maintain a higher level of consistency on certain subscales that appear prone to drift.

Providing reliability training via external TA trainers is more expensive than using internal TA trainers. Further research that examines the differences between these training methods could have significant implications for the cost of training practitioners in Ontario and elsewhere. Findings from this study support a shift to conduct our follow-up reliability exercises every 2 years, and we have continued to train internal TA trainers to expand this service within Ontario’s CAFAS user provider organizations.
